# Recent Trends in Plant Protein Complex Analysis in a Developmental Context

**DOI:** 10.3389/fpls.2018.00640

**Published:** 2018-05-15

**Authors:** Michiel Bontinck, Jelle Van Leene, Astrid Gadeyne, Bert De Rybel, Dominique Eeckhout, Hilde Nelissen, Geert De Jaeger

**Affiliations:** ^1^Department of Plant Biotechnology and Bioinformatics, Ghent University, Ghent, Belgium; ^2^Flanders Institute for Biotechnology, VIB-UGent Center for Plant Systems Biology, Ghent, Belgium

**Keywords:** plant development, affinity enrichment, tandem affinity purification, *Arabidopsis thaliana*, interactomics, proximity-dependent labeling

## Abstract

Because virtually all proteins interact with other proteins, studying protein–protein interactions (PPIs) is fundamental in understanding protein function. This is especially true when studying specific developmental processes, in which proteins often make developmental stage- or tissue specific interactions. However, studying these specific PPIs *in planta* can be challenging. One of the most widely adopted methods to study PPIs *in planta* is affinity purification coupled to mass spectrometry (AP/MS). Recent developments in the field of mass spectrometry have boosted applications of AP/MS in a developmental context. This review covers two main advancements in the field of affinity purification to study plant developmental processes: increasing the developmental resolution of the harvested tissues and moving from affinity purification to affinity enrichment. Furthermore, we discuss some new affinity purification approaches that have recently emerged and could have a profound impact on the future of protein interactome analysis in plants.

## Introduction

Proteins are the main workforce of biological systems and are involved in all aspects of life. They form the molecular machines responsible for basic cellular functions such as transcription, translation, metabolism and signal transduction, and for structural features such as the cytoskeleton. Extension of this basic protein repertoire allows more complex functions such as ensuring the developmental plan of an organism throughout its lifecycle or the deployment of mechanisms to sense environmental stimuli and generate the appropriate responses to these stimuli. These cellular responses can be short and reversible but they can also involve long-term, irreversible adaptations to the developmental plan of the organism to ensure its survival. To be able to carry out all these functions, proteins do not function on their own, but they rather interact with each other and are organized in networks of protein complexes and signaling cascades (Alberts, 1998). Studying these protein–protein interactions (PPIs) and exposing their intricate interaction networks are thus of fundamental importance to understand not only basic cellular processes but also complex developmental programs.

An elegant example of how PPIs are fundamental in determining plant development, is the specification of flower organ composition. Despite a huge diversity in different shapes, sizes and compositions, almost all flowers are built using only four specialized leaf types: sepals, petals, stamens, and carpels (Specht and Bartlett, 2009). The development of these different organ types is determined by the combinatorial activity of floral MADS-box TFs. According to the ABC model, A-type TFs specify sepal identity, A- and B-type together specify petal identity, B- and C-type together specify stamen identity and C-type alone specifies carpel identity (Coen and Meyerowitz, 1991). This model was later extended to the ABCDE model, also including D-type TFs for ovule specification and E-type TFs for proper development of all four organ types (Theißen et al., 2016; Thomson et al., 2017). Several lines of evidence have shown that these different types of TFs assemble into tetrameric protein complexes, so-called floral quartets (Bartlett, 2017). Although the exact downstream transcriptional networks of the floral quartets are still not fully understood (Thomson et al., 2017; Wils and Kaufmann, 2017), it is assumed that floral quartets with different compositions regulate alternative sets of target genes, which in turn results in the specification of a certain floral organ.

Several methods to test PPIs are currently available such as yeast Y2H (Mravec et al., 2011), co-immunoprecipitation followed by western blotting (co-IP) (Albrecht et al., 2012) or protein-fragment complementation assays such as BiFC (Ohad and Yalovsky, 2010). A clear characteristic of these methods is that they are binary methods, only allowing to test PPIs in a pairwise fashion. This often means that some prior knowledge is required to determine which combinations to test. Although massively multiplexed Y2H methods, such as CrY2H-seq (Trigg et al., 2017), allow screening for PPIs in a proteome-wide manner, these methods do not provide information on co-complex membership through indirect interaction. A complementary method that is more suited to study co-complex memberships is AP/MS. AP/MS is a collective name for different experimental approaches where a specific protein, referred to as the bait, is purified from a biological sample under near physiological conditions to keep PPIs intact. After purification, the co-purified proteins are identified by mass spectrometry. Because AP/MS does not require prior knowledge of the interaction partners, this technique is ideally suited to gain novel insights in the function of a protein of interest. An excellent example of this is the characterization of the TPLATE complex in Arabidopsis (Gadeyne et al., 2014). The TPLATE protein was originally discovered to be involved in cell plate anchoring during late cytokinesis, but the specific interaction with clathrin suggested a more general function in clathrin-mediated endocytosis (Van Damme et al., 2006). AP/MS experiments using TPLATE as bait protein revealed seven reproducibly interacting proteins of previously unknown function, which together form a stable multi-protein complex, subsequently called the TPLATE complex. Several of these interactions where subsequently validated using Y2H, BiFC, and co-IP. Furthermore, reciprocal AP/MS experiments using each of these seven interactors as bait proteins extended the network around TPLATE, revealing associations with members of the dynamin protein family, subunits of the Arabidopsis AP2 adaptor complex and of the clathrin scaffold, which ultimately led to the discovery of a plant-specific adaptation to clathrin-mediated endocytosis. Furthermore, the internalization and localization of auxin transport proteins and the brassinosteroid receptor BRI1, two well-known cargo proteins of clathrin-mediated endocytosis, are influenced by defects in the TPLATE complex, indicating the importance of TPLATE in plant development.

This review will cover the major steps and decisions to be made when setting up an AP/MS experiment. In doing so, we highlight two major trends in AP/MS experiments for studying plant development: increasing the developmental resolution of the harvested tissues and moving from affinity purification to affinity enrichment. We also discuss some recent technological advances in the field for which we anticipate that they could have a big impact on the future of AP/MS in plants.

## The Increase in Developmental Resolution

The first step in any AP/MS experiment encompasses generating a total protein extract from which the bait protein is to be purified. In theory, a bait protein can be purified from any tissue type where it is being expressed. This highlights one of the main advantages of using AP/MS, because it allows the identification of PPIs occurring *in vivo*, in a developmental context of choice, whereas most other binary methods such as Y2H often require ectopic expression systems such as yeast cells to express both bait and prey proteins.

### The Use of Cell Cultures

Cultured cells have traditionally been a popular source of biomass for AP/MS experiments. This is mainly due to their ease of transformation and high growth rates, which results in a fast, relatively cheap and nearly endless supply of biomass. The PSB-D cell suspension culture (Supplementary Table S1) has proven to be an excellent cell culture system for AP/MS purposes in plants. It is derived from the MM2d cell culture, which was originally generated from *Arabidopsis thaliana* Landsberg erecta stem explants (Menges and Murray, 2004). This PSB-D culture proliferates rapidly in the dark using sucrose as main energy source and has a 9C ploidy level leading to a diverse expression of proteins, often accumulating at high levels. The original protocol for AP/MS using this cell suspension culture demonstrated its value for studying PPIs regulating progression through the plant cell cycle (Van Leene et al., 2007), which subsequently resulted in a large cell cycle interactome that mapped the interaction networks surrounding approximately 100 core cell cycle proteins (Van Leene et al., 2010). Because in plants, post-embryonic growth is to a large extent determined by cell proliferation from various types of meristems, studying the cell cycle can provide valuable insights into organ development. Indeed, many proteins involved in cell cycle regulation and originating from the cell cycle interactome have been shown to influence final leaf size when their expression is altered (Blomme et al., 2014). For example, the elucidation of the cell cycle interactome led to the first description of SAMBA, a plant-specific regulator of the anaphase promoting complex/cyclosome (APC/C) E3 ligase (Eloy et al., 2012). SAMBA was found to be associated with the APC/C subunits APC3b, APC7, and APC10 (Van Leene et al., 2010). In reciprocal AP/MS experiments using SAMBA as a bait protein in cell cultures, almost all APC core complex subunits were identified as well as several known APC regulators (Eloy et al., 2012). Y2H validation of these results indicated that SAMBA specifically interacts with the APC/C by binding to the APC3b subunit. The role of SAMBA as an APC/C regulator in plant development was explored by examining the phenotype of *samba* knock-out mutants, which showed an increased size of seed, embryo, rosette area and root length. More specifically, SAMBA was suggested to inhibit cell proliferation during early plant development by targeting CYCLIN A2 for APC/C-mediated proteasomal degradation.

In addition to being an excellent model for dividing tissues, cell cultures have also been used to study protein complexes involved in other cellular processes such as hormone signaling (Geerinck et al., 2010; Pauwels et al., 2010; Fernández-Calvo et al., 2011; Antoni et al., 2013), secondary metabolism (Bassard et al., 2012) or intracellular trafficking (Nodzyński et al., 2013; Gadeyne et al., 2014). A particular advantage of using cell cultures is the ease with which these can be manipulated with chemicals such as hormones (Pauwels et al., 2010; Antoni et al., 2013) or synchronization compounds (Menges and Murray, 2002). Cell cultures from other organisms, such as rice (Zhong et al., 2003; Abe et al., 2008; Nallamilli et al., 2013) and tobacco (Nishikiori et al., 2011), have also been used, but these are far less popular than Arabidopsis cell cultures for AP/MS purposes. A major consideration to make with the use of cell cultures, however, is the fact that they are cultured callus tissues, which means they lack any kind of developmental context. This can lead to false-negative results when studying more specific developmental processes because these processes are not active in proliferative, cultured cells. Therefore, when studying plant development, the use of whole seedlings or, if technically possible, specific organs or even cell types is advised.

### The Use of Whole Plants and Organs

Several protocols describing the purification of protein complexes from Arabidopsis seedlings have been published over the years (Rohila et al., 2004; Rubio et al., 2005; Qi and Katagiri, 2009; Smaczniak et al., 2012b; Van Leene et al., 2015; Wendrich et al., 2017), resulting in a large collection of publications, a full overview of which is beyond the scope of this review. As a selected example, the identification of bZIP29-interacting proteins will be discussed here. bZIP29 was identified as a protein interacting with several cell cycle regulatory proteins in the cell cycle interactome, indicating a role in cell cycle regulation (Van Leene et al., 2010). bZIP29 is a member of the group I plant bZIP TFs, which have been mainly reported to play a role in vascular development and osmosensory responses. Therefore, the role of bZIP29 in plant development was further investigated and it was shown that bZIP29 is indeed predominantly expressed in proliferating tissues instead of vascular tissues (Van Leene et al., 2016). A dominant-negative version of bZIP29 resulted in a leaf phenotype characterized by a decreased cell number, which was compensated by an increased cell size. Defects were also detected in the gravitropic response and root meristem size. To identify interacting proteins, bZIP29 was purified through TAP from both cell cultures and seedlings. These experiments revealed several other group I bZIP TFs as interacting proteins (Van Leene et al., 2016), confirming the previous observation that bZIP TFs can form homo- and heterodimers (Tsugama et al., 2014). Interestingly, although the interacting bZIP proteins found in cell culture were also identified in seedlings, additional bZIP proteins were identified in seedlings. Furthermore, by comparing the expression profiles of the interacting bZIPs, certain tissue-specific heterodimers could be postulated. For example, overlapping the expression profile of bZIP69 with that of bZIP29 showed they were only expressed together in lateral root primordia, indicating this heterodimer could be specific for this tissue (Van Leene et al., 2016). This highlights the importance of incorporating transcript expression data into AP/MS data to aid in the biological interpretation of the data and hypothesize in which specific tissues the detected interactions might occur when using whole seedlings as biomass input instead of isolated tissues.

Because the sensitivity of mass spectrometers increased through the development of orbitrap mass spectrometers, the amount of biomass needed for successful purifications decreased. This allowed researchers to start dissecting organs from Arabidopsis seedlings and to zoom in on the developmental context they were interested in. For example, specifically harvesting the inflorescence meristems allowed Smaczniak et al. (2012a) to successfully identify the interaction networks surrounding five major homeotic MADS-domain proteins in inflorescence development. Here, representatives from the A, B, C, and E classes of the MADS-domain TFs were selected as bait protein for pull-down experiments, identifying many PPIs between different MADS-domain TFs, confirming the formation of floral quartets *in planta*. Furthermore, interactions with members of several other TF families were identified, indicating that floral quartets do not act alone but in concert with other TFs to achieve precise regulation of flower development. For example, APETALA1 (AP1), an A-type MADS-domain TF, was shown to interact with homeodomain TFs BELLRINGER (BLR), KNOTTED-LIKE 3 (KNAT3), and BEL1-LIKE HOMEODOMAIN 1 (BLH1), as well as with SUPPRESSOR OF OVEREXPRESSION OF CONSTANS 1 (SOC1), which is a major regulator of floral transition. Finally, also many links to chromatin remodeling were identified, showing that MADS-domain TFs can recruit the basic chromatin remodeling machinery to their target genes.

As one would expect, increasing the developmental resolution of the harvested starting material increases the resolution of the AP/MS experiment. A nice example to illustrate this is the series of AP/MS experiments that were performed using ANGUSTIFOLIA3/GRF-INTERACTING FACTOR1 (AN3/GIF1) as a bait protein. AN3 was originally identified as transcriptional coactivator interacting with the growth-regulating factor1 (GRF1) TF using Y2H screening (Kim and Kende, 2004). GRF1 is part of a plant-specific family of GRF TFs which have been shown to play key roles in stem and leaf development, but are also implicated in flower, seed and root development (Hoe Kim and Tsukaya, 2015; Omidbakhshfard et al., 2015). In many subsequent Y2H experiments, this interacting partnership extended to almost all members of the GRF and GIF protein families (Kim and Kende, 2004; Horiguchi et al., 2005; Liang et al., 2014). The molecular mechanism with which this GRF-GIF module exerts its function was first elucidated using TAP with AN3 as bait protein (Vercruyssen et al., 2014). Here, it was shown that AN3 interacts with SWI/SNF chromatin remodeling complexes. However, purifying AN3 from cell cultures and whole seedlings did not lead to the identification of GRF proteins that were already shown to be true interactors of AN3. On the contrary, when AN3 was purified using pull-down from developing inflorescences, both SWI/SNF complexes and GRF3 and GRF5 were identified (Debernardi et al., 2014). This indicates that the GRF-GIF interaction is a low-abundance interaction, which gets too diluted in a whole-seedling extract to be detected by mass spectrometry. Furthermore, a recent publication describes the TAP purification of AN3 from maize leaf tissues (Nelissen et al., 2015). The maize leaf is an excellent model to study the transition from cell division to cell expansion (Rymen et al., 2010), in which the GRF-GIF module also plays an important role (Hoe Kim and Tsukaya, 2015). Studying this developmental transition during leaf development in Arabidopsis is very challenging because the Arabidopsis leaf is about one mm in size at this developmental stage (Andriankaja et al., 2012). However, the developing maize leaf contains a growth zone at its base that is several cm in size and has a linear organization of dividing and expanding cells in the proliferation and expansion zones, respectively. These two tissues can easily be separated from each other and used as input material for AP/MS experiments to compare protein complex dynamics in these two developmental contexts (Rymen et al., 2010). When applying this experimental setup to AN3, it was shown that AN3 stably binds to the SWI/SNF chromatin remodeling complex throughout leaf development but interacts differently with GRF proteins in the cell division and cell expansion zones, which perfectly correlated with their underlying expression patterns. Furthermore, when AN3 protein complexes were purified from developing maize ears, other GRFs were identified compared to those isolated from leaf tissues (Nelissen et al., 2015). This example demonstrates the benefits of performing AP/MS experiments on specific organs or even sub-organ tissues as well as the need to transfer AP/MS to other model systems like certain crop species, which might be better suited, as was illustrated by the maize leaf. Similar complex purification strategies have been demonstrated for the rice leaf (Dedecker et al., 2016). The use of alternative model systems might also be required when specialized developmental processes are explored. For example, AP/MS has been transferred to the model legume *Medicago truncatula*, which is a well-known model species to study endosymbiotic interactions and specialized secondary metabolism (Goossens et al., 2016).

**FIGURE 1:**
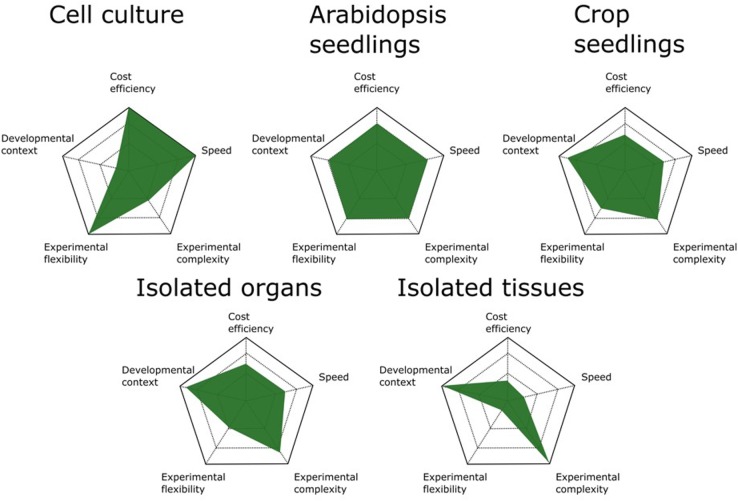
Comparison of available biomass sources for AP/MS experiments. The advantages and disadvantages of different biomass sources are scaled on five parameters. Cell cultures are the most cost- and time-efficient means of generating biomass for AP/MS experiments. They also allow the highest experimental flexibility because of the ease with which they are manipulated by chemical compounds. Arabidopsis seedlings are the best all-round biomass source, offering the best performance for standard AP/MS experiments. When studying more specific developmental processes, crop seedlings can be better suited. However, these are more time consuming and often allow less experimental flexibility because of their size. Although isolated organs and tissues offer the highest degree of developmental context, determining the optimal tissue and developmental stage to harvest biomass increases the complexity of the experimental setup and the time needed.

It is clear that increasing the developmental resolution of the harvested starting material furnishes AP/MS results with higher developmental content and allows digging deeper into the interactomes surrounding a bait protein. In principle, AP/MS can be performed on any tissue from any organism as long as a specific antibody for the bait protein is available or stable expression of a fusion protein can be achieved in the chosen tissue or organism. Because each biomass source has its advantages and disadvantages (**Figure 1**), the choice of which source material to use should be evaluated case by case and tailored to the experimental setup and biological question.

## An Introduction to Different Ap/Ms Approaches

**FIGURE 2:**
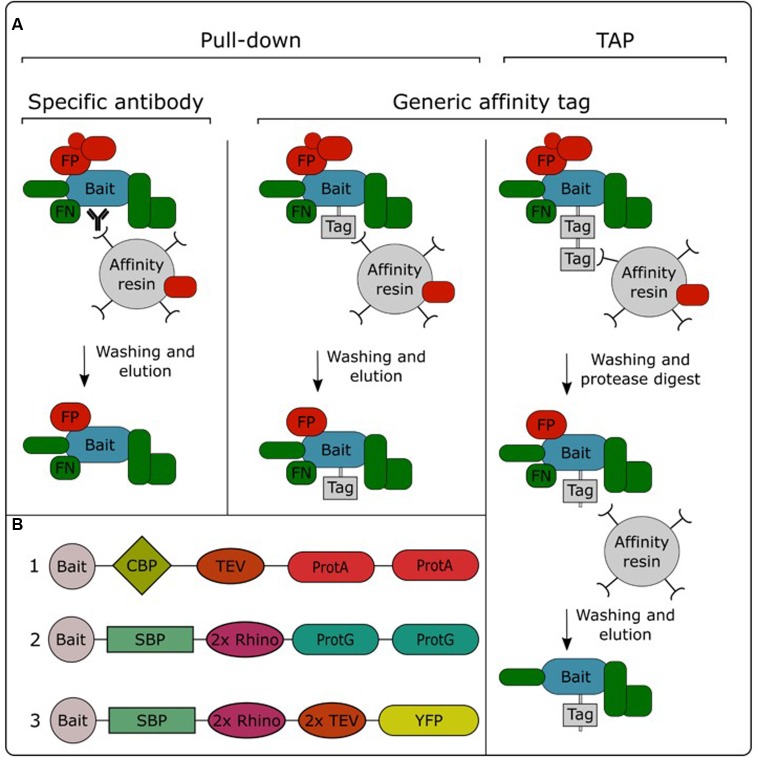
Overview of the different AP/MS approaches **(A)** and available TAP tags **(B)**. TAP tags: (1) TAPi tag; (2) GSrhino tag; (3) GS^yellow^ tag. CBP, calmodulin binding protein; FP, false positive; FN, false negative; ProtA, protein A domain; ProtG, protein G domain; 2x Rhino, double recognition site for the Rhinovirus 3C protease; TEV, recognition site for the tobacco etch virus protease; SBP, streptavidin-binding peptide; YFP, yellow fluorescent protein.

In order to be able to specifically purify a protein from a total protein extract, the bait protein needs to be captured and immobilized to an affinity resin. This allows non-interacting proteins to be washed away, while the interacting proteins stay immobilized. After removing non-interacting proteins, the interacting proteins can be eluted from the resin and identified using mass spectrometry (Morris et al., 2014). Purification of the bait protein can be performed in a single or double affinity purification protocol (**Figure 2A**). Single-step purifications typically use an antibody specific for the bait protein or a generic antibody against an affinity tag fused to the bait protein. These methods are therefore often called immunoprecipitation. However, single-step purification can also be performed without the need for specific antibodies, for example using the streptavidin-binding peptide-tag or a His-tag, which enable trapping with streptavidin or Ni2^+^ resins, respectively. We therefore prefer to use the term “pull-down” as a global term for single-step affinity purification. Alternatively, purification performed using a two-step affinity purification protocol is called TAP. In this approach, the bait protein is fused to a TAP-tag which contains two different affinity tags that are often separated by a protease cleavage site (**Figure 2B**). After immobilizing the bait protein using the first affinity domain and subsequent washing, this domain is cleaved off by incubation with a protease which specifically recognizes protein sequences linking both affinity domains. This cleavage step allows a gentle elution of the immobilized bait and exposes the second affinity domain for binding to the next affinity resin, permitting further removal of non-specific proteins by an additional washing step.

Pull-down experiments using an antibody specific for the bait protein allow purification of the endogenous protein, expressed from its native promoter. Therefore, there is no need to create transgenic lines expressing the bait protein fused to an affinity tag. Although this approach has been successfully applied in plants (Qi and Katagiri, 2009; König et al., 2014; Pertl-Obermeyer et al., 2014), it has not gained a lot of popularity in the field. This is mainly due to the limited amount of available plant protein antibodies, in combination with the fact that the development of specific antibodies can be a time-consuming and expensive effort. Furthermore, the specificity of these antibodies needs to be evaluated case by case, while generic antibodies are often specific and well characterized, allowing generic purification protocols. Therefore, the use of affinity tags is currently the standard practice in AP/MS experiments. A plethora of different affinity tags have been developed and evaluated over the years in plants (Dedecker et al., 2015). In general, fluorescent protein tags such as GFP are the most popular for pull-down experiments. This is mainly due to the availability of transgenic plant lines overexpressing GFP fusions in combination with the existence of high-quality anti-GFP antibodies. Moreover, the fluorescent protein tag can also be used to perform protein localization analysis. On the other hand, for TAP experiments, the TAPi tag (Rohila et al., 2004) and GS tag (Van Leene et al., 2008) are most widely adopted. A recently developed GS^yellow^ TAP tag (**Figure 2B**) combines the properties of both types of tags, combining the fluorescent protein Citrine YFP with the highly effective streptavidin-binding peptide tag into a double affinity tag (Besbrugge et al., 2018).

In an AP/MS experiment, the nature of the co-purified proteins is influenced by the purification method, choice of tag and tag position (Keilhauer et al., 2015). Adding a substantial protein domain to the bait protein, which is especially the case with large fluorescent protein tags, might cause interference with protein function. Therefore, it is advised to perform AP/MS experiments with both N- and C-terminal fusions of the tag to the bait protein.

## Dealing With False-Positive and False-Negative Results

The inherent nature of AP/MS protocols, during which cells are lysed, proteins are solubilized and bait proteins are purified, imposes the creation of both false-positive as well as false-negative interactions. By breaking the cells, the content of different cellular compartments is released into the protein extract, which allows the bait protein or its interactors to come into contact with proteins it could normally not interact with. On the other hand, false negatives arise during protein extraction and purification. By solubilizing the proteins in a protein extraction buffer, proteins are diluted, altering their concentration and binding kinetics. The subsequent purification of the bait protein requires binding to an affinity resin, removal of unbound proteins and iterative washing steps to reduce non-specific binding to the resin. These steps impose potential false-negative interactions because true interacting proteins that show weak interaction with the bait protein, are lost during the protein extract dilution step or during the different steps of the purification. In the following sections, we separate false-positive from false-negative results and discuss different approaches for dealing with them.

### False Positives: The Problem of Background Filtering

One of the biggest challenges in any AP/MS experiment is to discriminate between *bona fide* interacting proteins and non-specific background proteins. Non-specific background proteins are typically a mixture of false positives and true positives that interact with many unrelated proteins, such as proteins involved in translation, protein folding and transport. TAP was originally developed as a method to purify protein complexes at high purity under near-physiological conditions, minimizing the amount of background. However, with the increasing sensitivity of mass spectrometers, even protein complexes purified through TAP still contain a lot of background proteins and still require careful background filtering.

Traditionally, background filtering has been performed by mapping which proteins are identified in mock purifications or are reoccurring as co-purified proteins with different, unrelated bait proteins (Van Leene et al., 2015; Dedecker et al., 2016). This list of background proteins can then be used as a subtraction list to filter out background interactions, leaving only true and specific interactors. However, variations in the sample, purification protocol or sample preparation can significantly alter the protein identifications of an AP/MS experiment, hence also background identification. Therefore, single negative control experiments often fail to capture the complete set of contaminants. Large numbers of control purifications, negative controls or purifications of unrelated bait proteins, which have been performed under highly similar experimental conditions, need to be combined to achieve a comprehensive collection of background proteins. These background lists typically grow in size when more and more control or unrelated bait purifications are performed, or with the increased sensitivity of MS machines, potentially leading to false classification of proteins as non-specific for certain bait proteins. To deal with this problem, integration of semi-quantitative information on the abundance of a protein, i.e., based on normalized spectral abundance factors, has proven to be a valid method to recall true interactors from the background lists (Van Leene et al., 2015). Nevertheless, when compiled correctly, a background list offers a highly reliable and manually curated way of filtering the data. Online repositories of standardized negative control samples have been developed to overcome the need of individual researchers to perform large amounts of negative controls, for example the contaminant repository for affinity purification, i.e., the CRAPome (Mellacheruvu et al., 2013), and comprehensive background lists for Arabidopsis cell cultures, seedlings and rice cell cultures are publicly available (Van Leene et al., 2015; Dedecker et al., 2016; Supplementary Table S1).

However, when studying plant development, researchers often perform AP/MS experiments on specific tissue types or under specific experimental conditions, characterized by a unique set of expressed proteins. In these cases, comprehensive background lists, which have been developed for and under standardized experimental conditions, are insufficient. The emergence of quantitative proteomics has provided researchers with an alternative method to discriminate *bona fide* interacting proteins from background proteins without the need for large background lists. In quantitative AP/MS experiments, the quantity of proteins that co-purify with the bait proteins are compared to their quantities in a negative control sample. In this setup, background proteins will have a 1:1 ratio compared to the control, whereas true interacting proteins will be enriched. Many quantitative proteomics approaches have been developed and can be subdivided into chemical or metabolic labeling methods and label-free methods (Huang et al., 2016). In label-based quantitative proteomics, control and test samples are labeled with light and heavy isotopes. The samples are then mixed and measured in the same MS run, reducing experimental variation. Because identical peptides in different samples will be equally detected by the mass spectrometer, the difference in peak intensity between samples correlates with a difference in peptide abundance. Protein quantification is subsequently performed by calculating the intensity ratio of isotope-labeled peptide pairs. In label-free quantitative proteomics, control and test samples are measured in separate MS runs. Here, identical peptides are matched between runs based on their m/z value and retention time and protein quantification is performed by calculating the intensity ratio of matched peptides. Although chemical and metabolic labeling has been successfully applied in plants (Gouw et al., 2010; Minkoff et al., 2014; Liu et al., 2018), label-free methods have become the method of choice in plant quantitative AP/MS experiments (Podwojski et al., 2010; Zhu et al., 2010; Huang et al., 2016). The most-used algorithm for label-free quantification of AP/MS data is MaxQuant (Cox and Mann, 2008; Tyanova et al., 2016). An important note to make here is that discriminating between true and false interactors based on quantitative AP/MS data is a statistical process, meaning that proteins are considered to be true interactors when they are significantly enriched compared to the control. Therefore, the quality of the data analysis will improve with the size of the list of co-purified proteins. For this reason, single-step pull-down, which results in higher background levels compared to TAP and hence in a bigger dataset, actually works better in combination with quantitative data analysis compared to TAP samples. Because pull-down purifications rather enrich a bait protein instead of actually purifying it at high specificity, it is proposed that combining pull-down with label-free quantification should be renamed to affinity enrichment rather than affinity purification (Keilhauer et al., 2015).

### Putting Affinity Enrichment Into Practice

Affinity enrichment has been successfully performed in plants in numerous studies (Mravec et al., 2011; Smaczniak et al., 2012a,b; De Rybel et al., 2013; Debernardi et al., 2014; Nelissen et al., 2015; Née et al., 2017). Although a large variety of single affinity tags has been developed and used in the past (Dedecker et al., 2015), GFP has become the tag of choice for pull-down. A clear advantage of using fluorescence-based tags is that cellular protein expression and localization can be studied simultaneously using the same transgenic line. Moreover, these tags can guide researchers in their selection of tissues or developmental stages to be harvested. For example, this strategy was applied in the aforementioned study in which specific GRF TFs were found to be interacting with AN3 in inflorescences (Debernardi et al., 2014). Indeed, when visualizing the cellular localization of AN3-GFP, expressed from its endogenous promotor, high accumulation of AN3-GFP was detected in inflorescence meristems. This served as a good validation of the use of this tissue as starting material for an AP/MS experiment using AN3-GFP as bait protein. Another example is the investigation of the function of the bHLH TF TARGET OF MONOPTEROS5 (TMO5) in the establishment and maintenance of vascular tissue (De Rybel et al., 2013). Specific localization of TMO5-GFP was found in globular-stage embryos and mature roots. Therefore, siliques and seedling roots were used to purify TMO5-GFP and identify the bHLH TFs LHW and LHW-like2 as TMO5-interacting proteins. Reciprocal AP/MS using LHW as bait protein validated its interaction with TMO5 and additionally identified several TMO5-like proteins as interaction partners. Subsequent detailed exploration of these interaction profiles led to the description of a new bHLH heterodimer TF complex, which plays a crucial role in both the establishment of vascular tissues in the early embryo, as well as in maintaining the indeterminacy of this cell population in post-embryonic tissues.

Affinity enrichment has also been used to study seed dormancy in Arabidopsis (Née et al., 2017). This study was focused on the DELAY OF GERMINATION 1 (DOG1) protein. Although it was known that the amount of DOG1 is important for the time it takes to release seed dormancy and that this amount of DOG1 is dependent on environmental factors during seed maturation, no evidence toward the molecular function of DOG1 was reported. Using affinity enrichment for DOG1-YFP from four different seed conditions, dry versus 24 h imbibed for both dormant and non-dormant seeds, 184 interacting proteins were identified. Loss of dormancy drastically decreased the amount of interacting proteins, indicating loss of DOG1 activity after seed ripening. Further validation of specific interactions showed that DOG1 interacts with multiple phosphatases that have redundant but essential roles in the release of seed dormancy. Strikingly, similar phosphatases act as key negative regulators of the ABA signaling pathway, indicating that both signaling pathways controlling seed dormancy, converge on a set of distinct and partly overlapping protein phosphatases.

Recently, affinity enrichment protocols have been updated and further optimized for nuclear, cytoplasmic and membrane-associated protein complexes (Wendrich et al., 2017; Jamge et al., 2018).

### Validating AP/MS Results

Irrespective of which method was used for discriminating between *bona fide* interacting proteins and non-specific background proteins, additional validation of the identified PPIs remains important. For large-scale PPI networks generated by AP/MS, validation of at least a subset of the identified interactions is required to assess the quality of the data, whereas small-scale PPI networks allow a more extensive validation with multiple complementary methods to demonstrate the relevance of the identified interactions.

One of the most elegant ways of validating AP/MS results is performing reverse AP/MS experiments. Here, one or more of the identified interactors is used as bait protein to find reciprocal evidence for the PPIs. For example, the eight-subunit TPLATE complex was identified using TPLATE as bait, but all eight subunits could also be reciprocally identified using any of the TPLATE components as bait protein (Gadeyne et al., 2014). However, performing additional AP/MS experiments can become an expensive and time-consuming effort. As an alternative approach, reverse co-IP in the same genetic background as the AP/MS experiments can be applied. Relatively cheap and fast binary methods such as Y2H and BiFC are also popular methods for validating PPIs in a pairwise fashion. However, interactomes generated by binary methods and co-complex methods have been shown to overlap by less than 20% in yeast (Yu et al., 2008). Therefore, when validating AP/MS data with binary methods, one can expect a high rate of false negative results. For example, a subset of the cell cycle interactome was validated using the split-luciferase assay, resulting in only a 41% success rate. However, considering the low overlap between binary and co-complex data, this can actually be considered as a high success rate (Van Leene et al., 2010).

Finally, genetics can also be used to validate PPIs. For example, Huang et al. (2016) showed that phytochrome B (phyB) acts as a hub, connecting the circadian clock and red light signaling pathways. First, AP/MS using the evening complex component EARLY FLOWERING 4 (ELF4) as bait protein, led to the identification of other evening complex components, circadian clock proteins and several photoreceptors and light signaling regulators. Because phyB was the major associated photoreceptor, the experiment was repeated in a phyB mutant background. Here, it was shown that loss of phyB resulted in ELF4 losing its ability to interact with other clock and light signaling proteins, while its interactions with other evening complex components were still intact. In addition to validating the interaction between the evening complex and phyB, this experiment elegantly showed how coupling genetics to AP/MS can be used to identify sub-complexes and links between different signaling pathways.

### False Negatives: Detecting Transient or Weak Interactions

As mentioned earlier, AP/MS involves varying degrees of washing to remove background proteins and to increase the signal-to-noise ratio. However, during these washing steps, proteins that show weak or transient interaction with the bait protein are often lost. Indeed, it has been shown by comparing large-scale AP/MS datasets with Y2H datasets, that AP/MS datasets are enriched for stable interactions, whereas Y2H datasets are more enriched for transient interactions (Yu et al., 2008). A proposed solution to increase the chances of detecting transient or weak interactions are proximity-dependent labeling methods. With these methods, possible interacting proteins are labeled *in vivo* based on their proximity to a protein of interest. Labeled proteins can subsequently be affinity purified and identified using mass spectrometry. Several proximity-dependent labeling methods have been developed and reviewed elsewhere (Roux, 2013; Rees et al., 2015), but only a few have been used in plants.

A first method successfully applied in plants is *in vivo* crosslinking. Rohila et al. (2004) described a method to increase the recovery of interacting proteins in TAP protocols using *in vivo* crosslinking, which was used in several other AP/MS experiments (Qi and Katagiri, 2009; Pertl-Obermeyer et al., 2014; Bellati et al., 2016). A specific example of beneficial *in vivo* crosslinking is the purification of membrane proteins. Because these proteins contain highly hydrophobic patches embedded in the membranes, they require specific detergents for their solubilization, possibly resulting in the loss of interacting proteins. For this reason, it is notoriously hard to identify interacting proteins for membrane proteins, causing intrinsic membrane proteins to be clearly underrepresented in literature as bait proteins for AP/MS experiments in plants. In order to stabilize membrane protein interactions, Bellati et al. (2016) combined formaldehyde crosslinking with affinity enrichment to identify interacting proteins of the PIP1;2, and PIP2;1 aquaporines to gain additional insights into how root water transport is regulated. This work revealed a large-scale interactome comprising 436 and 388 interacting proteins of PIP1;2 and PIP2;1, respectively, of which 80% was shared between both proteins. This interactome provided significant insights into how PIP activity is regulated by processes such as intracellular trafficking, lipid signaling and also the activity of specific receptor-like kinases. As an alternative, protein complexes can also be stabilized *in vitro* during protein extraction, as exemplified by the usage of the reversible DSP crosslinker for analysis of the target of rapamycin interactome in Drosophila (Glatter et al., 2011).

A more recent promising example of proximity-dependent labeling plants is BioID, which is based on fusing a protein of interest to a mutant version of the *Escherichia coli* biotin ligase BirA (Roux et al., 2012). This mutant BirA biotinylates proteins that are within a 10-nm radius of the enzyme without the need for a specific recognition site on the target protein. Labeled proteins can subsequently be isolated using streptavidin-based affinity capture. Because the labeling is a covalent modification and because of the extremely strong biotin-streptavidin interaction, there is no need to maintain PPIs during the purification step, allowing harsh denaturing conditions during purification, which reduce background binding. BioID is very different from crosslinking in the sense that crosslinking merely provides a snapshot of the proteins in close proximity to the bait protein at the time of crosslinking. Instead, BioID generates a footprint of interactions made by the protein of interest over a period of time. Recently, an optimized version of the BioID protocol was published for rice protoplasts (Lin et al., 2017), showing its feasibility in plants, but also highlighting some drawbacks. For instance, because of the bacterial nature of the BirA protein, labeling is more efficient at higher temperature (Kim et al., 2016). However, growing plants at higher temperatures might give rise to temperature-induced activation of certain genes and consequently artificial interactions. Furthermore, to increase the efficiency of the BirA enzyme, exogenous biotin needs to be added to the growth medium (Lin et al., 2017). Also, control experiments need to be set up very carefully to filter out endogenously biotinylated proteins that contaminate the purification. Although proximity-dependent labeling methods merely provide information on which proteins are in close proximity to each other, it does not provide direct evidence for a physical interaction between these proteins, requiring further validation by independent PPI methods.

### Choosing the Right AP/MS Approach

**FIGURE 3:**
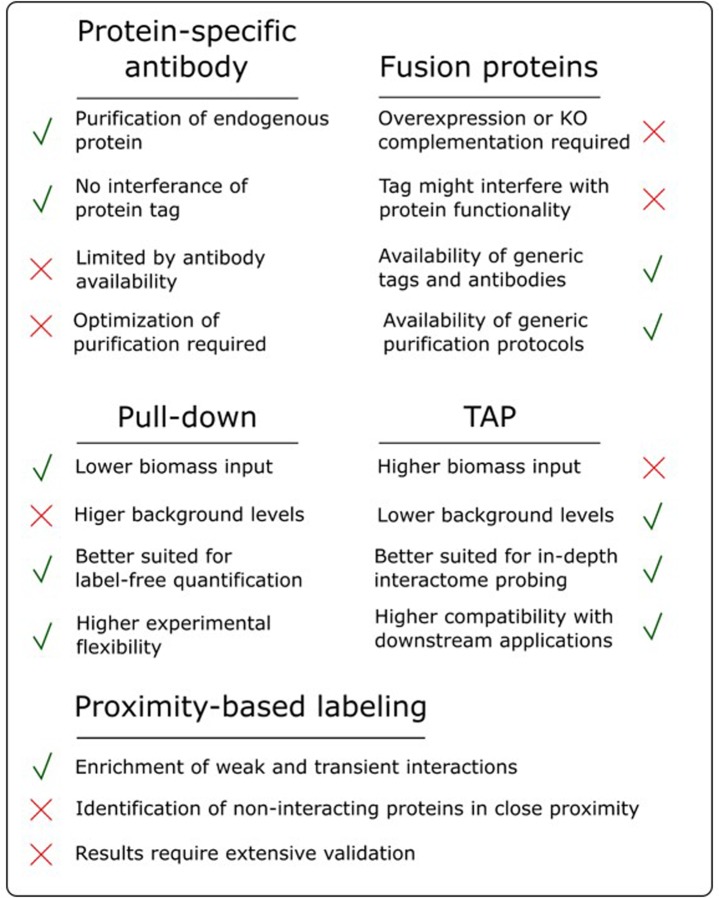
Overview of the advantages and disadvantages of various AP/MS approaches.

The success of an AP/MS experiment can be very bait-dependent and, as highlighted in the sections above, a lot of different parameters can influence the final results. Therefore, choosing the most suitable experimental setup can be a daunting task, often driven by intuition and prior experience. Having a good overview of the strengths and pitfalls of the available approaches is therefore instrumental for good decision making (**Figure 3**).

One of the most debated topics in this regard is the comparison of pull-down to TAP. As highlighted earlier, pull-down in combination with label-free quantification, referred to as affinity enrichment, is quickly gaining ground and might become the standard AP/MS method in plants. This popularity is mainly due to its high degree of flexibility toward changing experimental conditions, e.g., tissue types, treatments and model organism. Moreover, as described above, the lower signal-to-noise ratio in pull-down samples, compared to the high purity of TAP samples, is actually beneficial for subsequent analysis using label-free quantification (Keilhauer et al., 2015). Also, during a TAP purification, a substantial amount of bait protein is lost during the two-step purification protocol. Therefore, TAP generally requires more input material compared to pull-down. This can be especially problematic when harvesting a specific tissue type or experimental condition. Finally, because pull-down is a shorter, less exhaustive purification (**Figure 2**), it increases the chances of retaining weak or transient interactors, reducing the false negative rate. However, although affinity enrichment is quickly gaining ground in the AP/MS field, this does not mean that the TAP approach has become or will become obsolete. TAP was originally developed as a method to purify native protein complexes to high purity, which is still its major strength. The high signal-to-noise ratio of TAP samples compared to pull-down samples results in a higher chance of identifying sub-stoichiometrically interactors, which would otherwise be obscured by more abundant background proteins. Therefore, in an ideal setting, both pull-down and TAP/MS approaches should be applied to reach the full potential of what AP/MS can offer when studying protein interactomes. The high purity of TAP samples is also better suited for possible downstream applications such as *in vitro* activity assays using the purified complexes or separation of the purified sub-complexes using gel filtration.

## Endogenous Tagging

As highlighted earlier, pull-down using an antibody specific for the bait protein offers the most endogenous way of identifying interacting proteins using AP/MS. However, technical difficulties regarding these specific antibodies have limited their use. Therefore, most AP/MS experiments involve the use of fusion proteins which can be immobilized using generic antibodies. For the purification of a fusion protein to be successful, one of the key criteria is that the fusion protein outcompetes the endogenous protein for its interaction partners. Conceptually, knock-in targeting of the endogenous gene, thus keeping all endogenous regulatory elements intact, is the best way to tag a protein of interest. In simple biological systems like yeast, genes can readily be tagged at their endogenous locus by HR (Baudin et al., 1993). In higher organisms, however, endogenous tagging is much more challenging as a result of a much lower efficiency of HR (Puchta and Fauser, 2013). This is also true for plants, with the exception of the moss *Physcomitrella patens*, which displays a remarkable efficiency for integrating transgenes at a predefined locus through HR (Kamisugi et al., 2006). In Arabidopsis, two methods are used to circumvent inefficient HR. The first method, which is mainly used in cell cultures, is to overexpress the bait protein using a strong, constitutive promotor such as the Cauliflower mosaic virus 35S promotor. However, this overexpression method comes at the cost of obscuring the physiological gene dosage, which can affect protein folding and complex assembly, possibly leading to aberrant protein interactions and missing low-abundant interacting proteins because the non-complexed bait protein dominates the sample of purified proteins (Gibson et al., 2013). The second method uses transformation of the fusion protein under control of its endogenous promoter in a mutant background. In Arabidopsis, it is generally accepted that most regulatory sequences of a typical gene reside in the 2- to 3-kb region upstream of the start codon and the 0.5- to 1-kb region downstream of the stop codon (Tian et al., 2004). This is currently the method of choice in Arabidopsis because of the availability of large mutant collections and the fact that complementation of the mutant phenotype by the fusion protein can serve as a validation of the functionality of the fusion protein.

However, this method relies on the availability of a complete knock-out mutant, because any residual endogenous protein would compete with the fusion protein, as well as on the availability of the endogenous promoter. Moreover, even when the endogenous promotor is available, it is far from certain that it holds all necessary *cis*-regulatory elements. Therefore, it is not an exception that promotors lose part of their functionality when used in a transgene setting. As a consequence, researchers often prefer to use the constitutive 35S promotor to complement their mutant line, even if this might lead to the isolation of false-positive interactors. In species with less well-annotated genomes, it is even more challenging to predict which regulatory elements are responsible for proper expression of a gene and mutant collections are typically absent. Finally, this method of complementing mutants still relies on transforming the plants with a transgene, subjecting the T-DNA to position effects of the genomic regions surrounding the insertion site, which might influence the expression of the fusion protein.

An alternative method to achieve endogenous gene tagging while circumventing the need for HR in plants, is the recombineering-based gene tagging method (Zhou et al., 2011). This method uses bacterial artificial chromosomes containing large sections of a plant genome (tens of kb), in which a gene of interest can be tagged using the bacterial HR recombination system. Transforming these bacterial artificial chromosomes into mutant backgrounds effectively results in replacing the endogenous protein with a tagged counterpart, which is expressed with all its *cis*-regulatory elements intact. This method has been used to investigate protein localization during specific developmental processes such as cell differentiation in roots (Liberman et al., 2015; Moreno-Risueno et al., 2015) and hormone signaling (Péret et al., 2012; Zhang W. et al., 2013; Band et al., 2014), but has, to best of our knowledge, only been applied once for AP/MS purposes in plants (Lokdarshi et al., 2016). However, this method has the clear disadvantage that it not only introduces an endogenously tagged gene of interest but also a large genomic region surrounding this gene, possibly leading to changes in gene dosage.

Homologous recombination-based genome engineering in plants has been an active field of research for over 20 years now. Over time, scientists have used many technologies to induce double-strand DNA breaks, a prerequisite for HR, at specific sites in the genome such as zinc-finger nucleases, transcription activator-like effector nucleases and meganucleases (Steinert et al., 2016). Using these technologies, it was possible to modify endogenous genes for a variety of plant organisms such as Brachypodium, rice, maize, and tobacco (Mahfouz et al., 2011; Shan et al., 2013; Zhang Y. et al., 2013). However, the development of CRISPR/Cas genome engineering is clearly a game changer in this field. CRISPR/Cas allows unprecedented precision and efficiency in creating double-strand DNA breaks at a specific genomic locus and seems to work in almost every plant species tested so far. Significant advances in using CRISPR/Cas systems for endogenous tagging in plants have already been made, but efficiencies are still low and detection of HR events still requires extensive and laborious screening (Lokdarshi et al., 2016). It is expected, however, that it is only a matter of time before effective HR-mediated knock-in of a tag will be possible, which will further boost AP/MS in plants.

## Future Perspectives

As scientists dig deeper into understanding the PPI networks involved in plant development, the technologies they employ are continuously evolving. The constant increase in sensitivity of mass spectrometry has allowed researchers to start sampling more specific tissues, increasing the developmental resolution of the starting materials used for AP/MS experiments. One particular example of how the developmental resolution could be further increased is the integration of a method called ‘isolation of nuclei tagged in specific cell types’ (INTACT). INTACT uses a two-component system with a synthetic protein containing a nuclear targeting sequence, GFP and a biotin ligase recognition peptide, which gets biotinylated by a co-transformed BirA biotin ligase. Using spatially or temporally regulated promotors to drive the expression of this system, specific subtypes of nuclei within intact organs can be labeled with biotin and purified using a streptavidin resin (Deal and Henikoff, 2010). This method has already been applied to isolate cell-type specific nuclei from Arabidopsis roots (Deal and Henikoff, 2010; Foley et al., 2017), the early embryo (Palovaara et al., 2017), endosperm (Moreno-Romero et al., 2017) and tomato roots (Ron et al., 2014) and has recently been optimized for use in monocots (Reynoso et al., 2018). Although combining INTACT with AP/MS has not been reported yet, this technology would allow researchers to start mapping nuclear PPIs in a cell-type specific fashion.

AP/MS is also finding its way into other model organisms. In an ideal situation, scientists could choose their model system for its biological relevance and specific morphological and physiological characteristics or economic importance in function of the process one wants to study. In reality, however, Arabidopsis is the model of choice for AP/MS experiments in plants, mainly because of the efficiency at which this organism can be transformed. Currently, one of the biggest bottlenecks for applying AP/MS to any organism is the need for stable transformation with the fusion protein and thus, an efficient transformation protocol. However, the recent CRISPR/Cas revolution is causing tremendous amounts of resources to be directed toward optimizing transformation efficiencies, especially for crops (Altpeter et al., 2016). For example, it has recently been discovered that overexpression of the maize *Baby boom* (*Bbm*) and *Wuschel2* (*Wus2*) genes can stimulate transformation efficiencies in maize, sorghum, sugarcane, and rice (Lowe et al., 2016). Also, new technologies are being developed to circumvent the need for regeneration from tissue culture, which is still a major bottleneck in most transformation protocols. For example, magnetofection, a method using magnetic nanoparticles as DNA carriers, has recently been applied for the introduction of DNA into cotton pollen (Zhao et al., 2017). Subsequent pollination using the magnetofected pollen allowed the generation of transgenic seeds, resulting in stably transformed plants. Because plants generate large amounts of pollen, which can often also be easily collected, this method could also be easily transferred to other plant species.

In conclusion, AP/MS as a technology in plants has reached maturation, being routinely performed on Arabidopsis cell cultures and seedlings. We have exemplified that AP/MS has been instrumental for *in vivo* validation of interactions that were previously only detected using *in vitro* methods, as well as being a very useful tool for discovering new insights into the molecular function of proteins. As we have highlighted throughout this review, combining AP/MS with other molecular biology techniques such as high-resolution sampling, label-free quantitative proteomics or HR will allow researchers to tackle ever more challenging biological and developmental questions. In this way, AP/MS will undoubtedly benefit from future developments in other fields such as mass spectrometry, plant transformation and genome engineering. Thus, the future of AP/MS in studying plant development not merely lies in continuously improving AP/MS protocols, but in increasingly integrating AP/MS in more complex experimental workflows to maximize the success rate and developmental context of AP/MS results.

## Author Contributions

MB organized and wrote the manuscript. JVL, AG, DE, HN, BDR, and GDJ supervised and complemented the writing.

## Conflict of Interest Statement

The authors declare that the research was conducted in the absence of any commercial or financial relationships that could be construed as a potential conflict of interest.

## Abbreviations

AP/MS: affinity purification coupled to mass spectrometry
BiFC: bimolecular fluorescence complementation
co-IP: co-immunoprecipitation
HR: homologous recombination
PPI: protein–protein interaction
TAP: tandem affinity purification
TF: transcription factor
Y2H: two-hybrid

## References

[B1] Abe M. Fujiwara M. Kurotani K.-I. Yokoi S. Shimamoto K. (2008). Identification of dynamin as an interactor of rice GIGANTEA by tandem affinity purification (TAP). 49 420–432. 10.1093/Pcp/Pcn019 18296724

[B2] Alberts B. (1998). The cell as a collection of protein machines: preparing the next generation of molecular biologists. 92 291–294. 10.1016/S0092-8674(00)80922-8 9476889

[B3] Albrecht C. Boutrot F. Segonzac C. Schwessinger B. Gimenez-Ibanez S. Chinchilla D. (2012). Brassinosteroids inhibit pathogen-associated molecular pattern–triggered immune signaling independent of the receptor kinase BAK1. 109 303–308. 10.1073/pnas.1109921108 22087006 PMC3252947

[B4] Altpeter F. Springer N. M. Bartley L. E. Blechl A. E. Brutnell T. P. Citovsky V. (2016). Advancing crop transformation in the era of genome editing. 28 1510–1520. 10.1105/tpc.16.00196 27335450 PMC4981132

[B5] Andriankaja M. Dhondt S. De Bodt S. Vanhaeren H. Coppens F. De Milde L. (2012). Exit from proliferation during leaf development in *Arabidopsis thaliana*: a not-so-gradual process. 22 64–78. 10.1016/j.devcel.2011.11.011 22227310

[B6] Antoni R. Gonzalez-Guzman M. Rodriguez L. Peirats-Llobet M. Pizzio G. A. Fernandez M. A. (2013). PYRABACTIN RESISTANCE1-LIKE8 plays an important role for the regulation of abscisic acid signaling in root. 161 931–941. 10.1104/pp.112.208678 23370718 PMC3561030

[B7] Band L. R. Wells D. M. Fozard J. A. Ghetiu T. French A. P. Pound M. P. (2014). Systems analysis of auxin transport in the *Arabidopsis* root apex. 26 862–875. 10.1105/tpc.113.119495 24632533 PMC4001398

[B8] Bartlett M. E. (2017). Changing MADS-box transcription factor protein–protein interactions as a mechanism for generating floral morphological diversity. 57 1312–1321. 10.1093/icb/icx067 28992040

[B9] Bassard J.-E. Richert L. Geerinck J. Renault H. Duval F. Ullmann P. (2012). Protein-protein and protein-membrane associations in the lignin pathway. 24 4465–4482. 10.1105/tpc.112.102566 23175744 PMC3531846

[B10] Baudin A. Ozier-Kalogeropoulos O. Denouel A. Lacroute F. Cullin C. (1993). A simple and efficient method for direct gene deletion in *Saccharomyces cerevisiae*. 21 3329–3330. 10.1093/nar/21.14.3329 8341614 PMC309783

[B11] Bellati J. Champeyroux C. Hem S. Rofidal V. Krouk G. Maurel C. (2016). Novel aquaporin regulatory mechanisms revealed by interactomics. 15 3473–3487. 10.1074/mcp.M116.060087 27609422 PMC5098044

[B12] Besbrugge N. Van Leene J. Eeckhout D. Cannoot B. Kulkarni S. R. De Winne N. (2018). GSyellow, a swiss-knife tag for functional protein analysis in monocot and dicot plants. [Epub ahead of print]. 10.1104/pp.18.00175 29678859 PMC6001315

[B13] Blomme J. Inzé D. Gonzalez N. (2014). The cell-cycle interactome: a source of growth regulators? 65 2715–2730. 10.1093/jxb/ert388 24298000

[B14] Coen E. S. Meyerowitz E. M. (1991). The war of the whorls: genetic interactions controlling flower development. 353 31–37. 10.1038/353031a0 1715520

[B15] Cox J. Mann M. (2008). MaxQuant enables high peptide identification rates, individualized p.p.b.-range mass accuracies and proteome-wide protein quantification. 26 1367–1372. 10.1038/nbt.1511 19029910

[B16] De Rybel B. Möller B. Yoshida S. Grabowicz I. De Reuille P. B. Boeren S. (2013). A bHLH complex controls embryonic vascular tissue establishment and indeterminate growth in *Arabidopsis*. 24 426–437. 10.1016/j.devcel.2012.12.013 23415953

[B17] Deal R. B. Henikoff S. (2010). A simple method for gene expression and chromatin profiling of individual cell types within a tissue. 18 1030–1040. 10.1016/j.devcel.2010.05.013 20627084 PMC2905389

[B18] Debernardi J. M. Mecchia M. A. Vercruyssen L. Smaczniak C. Kaufmann K. Inzé D. (2014). Post-transcriptional control of GRF transcription factors by microRNA miR396 and GIF co-activator affects leaf size and longevity. 79 413–426. 10.1111/tpj.12567 24888433

[B19] Dedecker M. Van Leene J. De Jaeger G. (2015). Unravelling plant molecular machineries through affinity purification coupled to mass spectrometry. 24 1–9. 10.1016/j.pbi.2015.01.001 25603557

[B20] Dedecker M. Van Leene J. De Winne N. Eeckhout D. Persiau G. Van De Slijke E. (2016). Transferring an optimized TAP-toolbox for the isolation of protein complexes to a portfolio of rice tissues. 91 341–354. 10.1007/s11103-016-0471-x 27003905

[B21] Eloy N. B. Gonzalez N. Van Leene J. Maleux K. Vanhaeren H. De Milde L. (2012). SAMBA, a plant-specific anaphase-promoting complex/cyclosome regulator is involved in early development and A-type cyclin stabilization. 109 13853–13858. 10.1073/pnas.1211418109 22869741 PMC3427114

[B22] Fernández-Calvo P. Chini A. Fernández-Barbero G. Chico J.-M. Gimenez-Ibanez S. Geerinck J. (2011). The Arabidopsis bHLH transcription factors MYC3 and MYC4 are targets of JAZ repressors and act additively with MYC2 in the activation of jasmonate responses. 23 701–715. 10.1105/tpc.110.080788 21335373 PMC3077776

[B23] Foley S. W. Gosai S. J. Wang D. Selamoglu N. Sollitti A. C. Köster T. (2017). A global view of RNA-protein interactions identifies post-transcriptional regulators of root hair cell fate. 41 204.e5–220.e5. 10.1016/j.devcel.2017.03.018 28441533 PMC5605909

[B24] Gadeyne A. Sánchez-Rodríguez C. Vanneste S. Di Rubbo S. Zauber H. Vanneste K. (2014). The TPLATE adaptor complex drives clathrin-mediated endocytosis in plants. 156 691–704. 10.1016/j.cell.2014.01.039 24529374

[B25] Geerinck J. Pauwels L. De Jaeger G. Goossens A. (2010). Dissection of the one-MegaDalton JAZ1 protein complex. 5 1039–1041. 10.4161/psb.5.8.12338 20671423 PMC3115192

[B26] Gibson T. J. Seiler M. Veitia R. A. (2013). The transience of transient overexpression. 10 715–721. 10.1038/nmeth.2534 23900254

[B27] Glatter T. Schittenhelm R. B. Rinner O. Roguska K. Wepf A. Jünger M. A. (2011). Modularity and hormone sensitivity of the *Drosophila melanogaster* insulin receptor/target of rapamycin interaction proteome. 7:547. 10.1038/msb.2011.79 22068330 PMC3261712

[B28] Goossens J. De Geyter N. Walton A. Eeckhout D. Mertens J. Pollier J. (2016). Isolation of protein complexes from the model legume *Medicago truncatula* by tandem affinity purification in hairy root cultures. 88 476–489. 10.1111/tpj.13258 27377668

[B29] Gouw J. W. Krijgsveld J. Heck A. J. R. (2010). Quantitative proteomics by metabolic labeling of model organisms. 9 11–24. 10.1074/mcp.R900001-MCP200 19955089 PMC2808257

[B30] Hoe Kim J. Tsukaya H. (2015). Regulation of plant growth and development by the GROWTH-REGULATING FACTOR and GRF-INTERACTING FACTOR duo. 66 6093–6107. 10.1093/jxb/erv349 26160584

[B31] Horiguchi G. Kim G.-T. Tsukaya H. (2005). The transcription factor AtGRF5 and the transcription coactivator AN3 regulate cell proliferation in leaf primordia of *Arabidopsis thaliana*. 43 68–78. 10.1111/j.1365-313X.2005.02429.x 15960617

[B32] Huang H. Alvarez S. Bindbeutel R. Shen Z. Naldrett M. J. Evans B. S. (2016). Identification of evening complex associated proteins in *Arabidopsis thaliana* by affinity purification and mass spectrometry. 15 201–217. 10.1074/mcp.M115.054064 26545401 PMC4762519

[B33] Jamge S. Angenent G. C. Bemer M. (2018). Identification of in planta protein–protein interactions using IP-MS. 1675 315–329. 10.1007/978-1-4939-7318-7_18 29052199

[B34] Kamisugi Y. Schlink K. Rensing S. A. Schween G. Von Stackelberg M. Cuming A. C. (2006). The mechanism of gene targeting in *Physcomitrella patens*: homologous recombination, concatenation and multiple integration. 34 6205–6214. 10.1093/nar/gkl832 17090599 PMC1693892

[B35] Keilhauer E. C. Hein M. Y. Mann M. (2015). Accurate protein complex retrieval by affinity enrichment mass spectrometry (AE-MS) rather than affinity purification mass spectrometry (AP-MS). 14 120–135. 10.1074/mcp.M114.041012 25363814 PMC4288248

[B36] Kim D. I. Jensen S. C. Noble K. A. Birendra K. C. Roux K. H. Motamedchaboki K. (2016). An improved smaller biotin ligase for BioID proximity labeling. 27 1188–1196. 10.1091/mbc.E15-12-0844 26912792 PMC4831873

[B37] Kim J. H. Kende H. (2004). A transcriptional coactivator, AtGIF1, is involved in regulating leaf growth and morphology in *Arabidopsis*. 101 13374–13379. 10.1073/pnas.0405450101 15326298 PMC516574

[B38] König A.-C. Hartl M. Pham P. A. Laxa M. Boersema P. J. Orwat A. (2014). The Arabidopsis class II sirtuin is a lysine deacetylase and interacts with mitochondrial energy metabolism. 164 1401–1414. 10.1104/pp.113.232496 24424322 PMC3938629

[B39] Liang G. He H. Li Y. Wang F. Yu D. (2014). Molecular mechanism of microRNA396 mediating pistil development in Arabidopsis. 164 249–258. 10.1104/pp.113.225144 24285851 PMC3875806

[B40] Liberman L. M. Sparks E. E. Moreno-Risueno M. A. Petricka J. J. Benfey P. N. (2015). MYB36 regulates the transition from proliferation to differentiation in the Arabidopsis root. 112 12099–12104. 10.1073/pnas.1515576112 26371322 PMC4593085

[B41] Lin Q. Zhou Z. Luo W. Fang M. Li M. Li H. (2017). Screening of proximal and interacting proteins in rice protoplasts by proximity-dependent biotinylation. 8:749. 10.3389/fpls.2017.00749 28553299 PMC5427108

[B42] Liu S. Yu F. Yang Z. Wang T. Xiong H. Chang C. (2018). Establishment of dimethyl labelling-based quantitative acetylproteomics in Arabidopsis. 10.1074/mcp.RA117.000530 [Epub ahead of print]. 29440448 PMC5930405

[B43] Lokdarshi A. Conner W. C. Mcclintock C. Li T. Roberts D. M. (2016). Arabidopsis CML38, a calcium sensor that localizes to ribonucleoprotein complexes under hypoxia stress. 170 1046–1059. 10.1104/pp.15.01407 26634999 PMC4734562

[B44] Lowe K. Wu E. Wang N. Hoerster G. Hastings C. Cho M.-J. (2016). Morphogenic regulators Baby boom and Wuschel improve monocot transformation. 28 1998–2015. 10.1105/tpc.16.00124 27600536 PMC5059793

[B45] Mahfouz M. M. Li L. Shamimuzzaman M. Wibowo A. Fang X. Zhu J.-K. (2011). De novo-engineered transcription activator-like effector (TALE) hybrid nuclease with novel DNA binding specificity creates double-strand breaks. 108 2623–2628. 10.1073/pnas.1019533108 21262818 PMC3038751

[B46] Mellacheruvu D. Wright Z. Couzens A. L. Lambert J.-P. St-Denis N. A. Li T. (2013). The CRAPome: a contaminant repository for affinity purification-mass spectrometry data. 10 730–736. 10.1038/Nmeth.2557 23921808 PMC3773500

[B47] Menges M. Murray J. A. H. (2002). Synchronous *Arabidopsis* suspension cultures for analysis of cell-cycle gene activity. 30 203–212. 10.1046/j.1365-313X.2002.01274.x 12000456

[B48] Menges M. Murray J. A. H. (2004). Cryopreservation of transformed and wild-type *Arabidopsis* and tobacco cell suspension cultures. 37 635–644. 10.1046/j.1365-313X.2003.01980.x 14756764

[B49] Minkoff B. B. Burch H. L. Sussman M. R. (2014). A pipeline for 15N metabolic labeling and phosphoproteome analysis in *Arabidopsis thaliana*. 1062 353–379. 10.1007/978-1-62703-580-4_19 24057376

[B50] Moreno-Risueno M. A. Sozzani R. Yardımcı G. G. Petricka J. J. Vernoux T. Blilou I. (2015). Transcriptional control of tissue formation throughout root development. 350 426–430. 10.1126/science.aad1171 26494755 PMC4855878

[B51] Moreno-Romero J. Santos-González J. Hennig L. Köhler C. (2017). Applying the INTACT method to purify endosperm nuclei and to generate parental-specific epigenome profiles. 12 238–254. 10.1038/nprot.2016.167 28055034

[B52] Morris J. H. Knudsen G. M. Verschueren E. Johnson J. R. Cimermancic P. Greninger A. L. (2014). Affinity purification–mass spectrometry and network analysis to understand protein-protein interactions. 9 2539–2554. 10.1038/nprot.2014.164 25275790 PMC4332878

[B53] Mravec J. Petrášek J. Li N. Boeren S. Karlova R. Kitakura S. (2011). Cell plate restricted association of DRP1A and PIN proteins is required for cell polarity establishment in *Arabidopsis*. 21 1055–1060. 10.1016/j.cub.2011.05.018 21658946

[B54] Nallamilli B. R. R. Zhang J. Mujahid H. Malone B. M. Bridges S. M. Peng Z. (2013). Polycomb group gene OsFIE2 regulates rice (*Oryza sativa*) seed development and grain filling via a mechanism distinct from *Arabidopsis*. 9:e1003322. 10.1371/journal.pgen.1003322 23505380 PMC3591265

[B55] Née G. Kramer K. Nakabayashi K. Yuan B. Xiang Y. Miatton E. (2017). DELAY OF GERMINATION1 requires PP2C phosphatases of the ABA signalling pathway to control seed dormancy. 8:72. 10.1038/s41467-017-00113-6 28706187 PMC5509711

[B56] Nelissen H. Eeckhout D. Demuynck K. Persiau G. Walton A. Van Bel M. (2015). Dynamic changes in ANGUSTIFOLIA3 complex composition reveal a growth regulatory mechanism in the maize leaf. 27 1605–1619. 10.1105/tpc.15.00269 26036253 PMC4498210

[B57] Nishikiori M. Mori M. Dohi K. Okamura H. Katoh E. Naito S. (2011). A host small GTP-binding protein ARL8 plays crucial roles in tobamovirus RNA replication. 7:e1002409. 10.1371/journal.ppat.1002409 22174675 PMC3234234

[B58] Nodzyński T. Feraru M. I. Hirsch S. De Rycke R. Niculaes C. Boerjan W. (2013). Retromer subunits VPS35A and VPS29 mediate prevacuolar compartment (PVC) function in *Arabidopsis*. 6 1849–1862. 10.1093/mp/sst044 23770835

[B59] Ohad N. Yalovsky S. (2010). Utilizing bimolecular fluorescence complementation (BiFC) to assay protein–protein interaction in plants. 655 347–358. 10.1007/978-1-60761-765-5_23 20734272

[B60] Omidbakhshfard M. Proost S. Fujikura U. Mueller-Roeber B. (2015). Growth-regulating factors (GRFs): a small transcription factor family with important functions in plant biology. 8 998–1010. 10.1016/j.molp.2015.01.013 25620770

[B61] Palovaara J. Saiga S. Wendrich J. R. Van ’T Wout Hofland N. Van Schayck J. P. Hater F. (2017). Transcriptome dynamics revealed by a gene expression atlas of the early *Arabidopsis* embryo. 3 894–904. 10.1038/s41477-017-0035-3 29116234 PMC5687563

[B62] Pauwels L. Barbero G. F. Geerinck J. Tilleman S. Grunewald W. Cuéllar Pérez A. (2010). NINJA connects the co-repressor TOPLESS to jasmonate signalling. 464 788–791. 10.1038/nature08854 20360743 PMC2849182

[B63] Péret B. Swarup K. Ferguson A. Seth M. Yang Y. Dhondt S. (2012). AUX/LAX genes encode a family of auxin influx transporters that perform distinct functions during *Arabidopsis* development. 24 2874–2885. 10.1105/tpc.112.097766 22773749 PMC3426120

[B64] Pertl-Obermeyer H. Schulze W. X. Obermeyer G. (2014). In vivo cross-linking combined with mass spectrometry analysis reveals receptor-like kinases and Ca2+ signalling proteins as putative interaction partners of pollen plasma membrane H+ ATPases. 108 17–29. 10.1016/j.jprot.2014.05.001 24824344

[B65] Podwojski K. Eisenacher M. Kohl M. Turewicz M. Meyer H. E. Rahnenführer J. (2010). Peek a peak: a glance at statistics for quantitative label-free proteomics. 7 249–261. 10.1586/epr.09.107 20377391

[B66] Puchta H. Fauser F. (2013). Gene targeting in plants: 25 years later. 57 629–637. 10.1387/ijdb.130194hp 24166445

[B67] Qi Y. Katagiri F. (2009). Purification of low-abundance Arabidopsis plasma-membrane protein complexes and identification of candidate components. 57 932–944. 10.1111/j.1365-313X.2008.03736.x 19000159

[B68] Rees J. S. Li X.-W. Perrett S. Lilley K. S. Jackson A. P. (2015). Protein neighbors and proximity proteomics. 14 2848–2856. 10.1074/mcp.R115.052902 26355100 PMC4638030

[B69] Reynoso M. Pauluzzi G. Kajala K. Cabanlit S. Velasco J. Bazin J. (2018). Nuclear transcriptomes at high resolution using retooled INTACT. 176 270–281. 10.1104/pp.17.00688 28956755 PMC5761756

[B70] Rohila J. S. Chen M. Cerny R. Fromm M. E. (2004). Improved tandem affinity purification tag and methods for isolation of protein heterocomplexes from plants. 38 172–181. 10.1111/j.1365-313X.2004.02031.x 15053770

[B71] Ron M. Kajala K. Pauluzzi G. Wang D. Reynoso M. A. Zumstein K. (2014). Hairy root transformation using *Agrobacterium rhizogenes* as a tool for exploring cell type-specific gene expression and function using tomato as a model. 166 455–469. 10.1104/pp.114.239392 24868032 PMC4213079

[B72] Roux K. J. (2013). Marked by association: techniques for proximity-dependent labeling of proteins in eukaryotic cells. 70 3657–3664. 10.1007/s00018-013-1287-3 23420482 PMC11113768

[B73] Roux K. J. Kim D. I. Raida M. Burke B. (2012). A promiscuous biotin ligase fusion protein identifies proximal and interacting proteins in mammalian cells. 196 801–810. 10.1083/jcb.201112098 22412018 PMC3308701

[B74] Rubio V. Shen Y. Saijo Y. Liu Y. Gusmaroli G. Dinesh-Kumar S. P. (2005). An alternative tandem affinity purification strategy applied to Arabidopsis protein complex isolation. 41 767–778. 10.1111/j.1365-313X.2004.02328.x 15703063

[B75] Rymen B. Coppens F. Dhondt S. Fiorani F. Beemster G. T. S. (2010). Kinematic analysis of cell division and expansion. 655 203–227. 10.1007/978-1-60761-765-5_14 20734263

[B76] Shan Q. Wang Y. Chen K. Liang Z. Li J. Zhang Y. (2013). Rapid and efficient gene modification in rice and *Brachypodium* using TALENs. 6 1365–1368. 10.1093/mp/sss162 23288864 PMC3968307

[B77] Smaczniak C. Immink R. G. H. Muiño J. M. Blanvillain R. Busscher M. Busscher-Lange J. (2012a). Characterization of MADS-domain transcription factor complexes in Arabidopsis flower development. 109 1560–1565. 10.1073/pnas.1112871109 22238427 PMC3277181

[B78] Smaczniak C. Li N. Boeren S. America T. Van Dongen W. Goerdayal S. S. (2012b). Proteomics-based identification of low-abundance signaling and regulatory protein complexes in native plant tissues. 7 2144–2158. 10.1038/nprot.2012.129 23196971

[B79] Specht C. D. Bartlett M. E. (2009). Flower evolution: the origin and subsequent diversification of the angiosperm flower. 40 217–243. 10.1146/annurev.ecolsys.110308.120203

[B80] Steinert J. Schiml S. Puchta H. (2016). Homology-based double-strand break-induced genome engineering in plants. 35 1429–1438. 10.1007/s00299-016-1981-3 27084537

[B81] Theißen G. Melzer R. Rümpler F. (2016). MADS-domain transcription factors and the floral quartet model of flower development: linking plant development and evolution. 143 3259–3271. 10.1242/dev.134080 27624831

[B82] Thomson B. Zheng B. Wellmer F. (2017). Floral organogenesis: when knowing your ABCs is not enough. 173 56–64. 10.1104/pp.16.01288 27789738 PMC5210729

[B83] Tian G.-W. Mohanty A. Chary S. N. Li S. Paap B. Drakakaki G. (2004). High-throughput fluorescent tagging of full-length Arabidopsis gene products in planta. 135 25–38. 10.1104/pp.104.040139 15141064 PMC429330

[B84] Trigg S. A. Garza R. M. Macwilliams A. Nery J. R. Bartlett A. Castanon R. (2017). CrY2H-seq: a massively multiplexed assay for deep-coverage interactome mapping. 14 819–825. 10.1038/nmeth.4343 28650476 PMC5564216

[B85] Tsugama D. Liu S. Takano T. (2014). Analysis of functions of VIP1 and its close homologs in osmosensory responses of *Arabidopsis thaliana*. 9:e103930. 10.1371/journal.pone.0103930 25093810 PMC4122391

[B86] Tyanova S. Temu T. Cox J. (2016). The MaxQuant computational platform for mass spectrometry-based shotgun proteomics. 11 2301–2319. 10.1038/nprot.2016.136 27809316

[B87] Van Damme D. Coutuer S. De Rycke R. Bouget F.-Y. Inzé D. Geelen D. (2006). Somatic cytokinesis and pollen maturation in *Arabidopsis* depend on TPLATE, which has domains similar to coat proteins. 18 3502–3518. 10.1105/tpc.106.040923 17189342 PMC1785392

[B88] Van Leene J. Blomme J. Kulkarni S. R. Cannoot B. De Winne N. Eeckhout D. (2016). Functional characterization of the *Arabidopsis* transcription factor bZIP29 reveals its role in leaf and root development. 67 5825–5840. 10.1093/jxb/erw347 27660483 PMC5066499

[B89] Van Leene J. Eeckhout D. Cannoot B. De Winne N. Persiau G. Van De Slijke E. (2015). An improved toolbox to unravel the plant cellular machinery by tandem affinity purification of *Arabidopsis* protein complexes. 10 169–187. 10.1038/nprot.2014.199 25521792

[B90] Van Leene J. Hollunder J. Eeckhout D. Persiau G. Van De Slijke E. Stals H. (2010). Targeted interactomics reveals a complex core cell cycle machinery in *Arabidopsis thaliana*. 6:397. 10.1038/msb.2010.53 20706207 PMC2950081

[B91] Van Leene J. Stals H. Eeckhout D. Persiau G. Van De Slijke E. Van Isterdael G. (2007). A tandem affinity purification-based technology platform to study the cell cycle interactome in *Arabidopsis thaliana*. 6 1226–1238. 10.1074/mcp.M700078-MCP200 17426018

[B92] Van Leene J. Witters E. Inzé D. De Jaeger G. (2008). Boosting tandem affinity purification of plant protein complexes. 13 517–520. 10.1016/j.tplants.2008.08.002 18771946

[B93] Vercruyssen L. Verkest A. Gonzalez N. Heyndrickx K. S. Eeckhout D. Han S.-K. (2014). ANGUSTIFOLIA3 binds to SWI/SNF chromatin remodeling complexes to regulate transcription during *Arabidopsis* leaf development. 26 210–229. 10.1105/tpc.113.115907 24443518 PMC3963571

[B94] Wendrich J. R. Boeren S. Möller B. K. Weijers D. De Rybel B. (2017). In vivo identification of plant protein complexes using IP-MS/MS. 1497 147–158. 10.1007/978-1-4939-6469-7_14 27864765

[B95] Wils C. R. Kaufmann K. (2017). Gene-regulatory networks controlling inflorescence and flower development in *Arabidopsis thaliana*. 1860 95–105. 10.1016/j.bbagrm.2016.07.014 27487457

[B96] Yu H. Braun P. Yildirim M. A. Lemmens I. Venkatesan K. Sahalie J. (2008). High-quality binary protein interaction map of the yeast interactome network. 322 104–110. 10.1126/science.1158684 18719252 PMC2746753

[B97] Zhang W. Swarup R. Bennett M. Schaller G. E. Kieber J. J. (2013). Cytokinin induces cell division in the quiescent center of the *Arabidopsis* root apical meristem. 23 1979–1989. 10.1016/j.cub.2013.08.008 24120642

[B98] Zhang Y. Zhang F. Li X. Baller J. A. Qi Y. Starker C. G. (2013). Transcription activator-like effector nucleases enable efficient plant genome engineering. 161 20–27. 10.1104/pp.112.205179 23124327 PMC3532252

[B99] Zhao X. Meng Z. Wang Y. Chen W. Sun C. Cui B. (2017). Pollen magnetofection for genetic modification with magnetic nanoparticles as gene carriers. 3 956–964. 10.1038/s41477-017-0063-z 29180813

[B100] Zhong J. Haynes P. A. Zhang S. Yang X. Andon N. L. Eckert D. (2003). Development of a system for the study of protein-protein interactions in planta: characterization of a TATA-box binding protein complex in *Oryza sativa*. 2 514–522. 10.1021/pr034023z 14582648

[B101] Zhou R. Benavente L. M. Stepanova A. N. Alonso J. M. (2011). A recombineering-based gene tagging system for Arabidopsis. 66 712–723. 10.1111/j.1365-313X.2011.04524.x 21294796

[B102] Zhu W. Smith J. W. Huang C.-M. (2010). Mass spectrometry-based label-free quantitative proteomics. 2010:840518. 10.1155/2010/840518 19911078 PMC2775274

